# Erratum to “The Role of Parenting Behaviors in the Bidirectional and Intergenerational Transmission of Depression and Anxiety Between Parents and Early Adolescent Youth”

**DOI:** 10.1155/da/9827354

**Published:** 2025-02-22

**Authors:** Carly J. Johnco, Natasha R. Magson, Jasmine Fardouly, Ella L. Oar, Miriam K. Forbes, Cele Richardson, Ronald M. Rapee

**Affiliations:** ^1^Department of Psychology, Centre for Emotional Health, Macquarie University, Sydney, New South Wales, Australia; ^2^School of Psychological Science, Centre for Sleep Science, University of Western Australia, Perth, Western Australia, Australia

In the article titled “The Role of Parenting Behaviors in the Bidirectional and Intergenerational Transmission of Depression and Anxiety Between Parents and Early Adolescent Youth” [1], there were errors in Figure 7a,b. The figure should show the incorrect panel in Figure 7.

The error was introduced during the production process of the article, and Wiley apologizes for causing this error in the article.

Figure 7a,b should be corrected as follows and is listed as Figure 1.

## Figures and Tables

**Figure 1:**
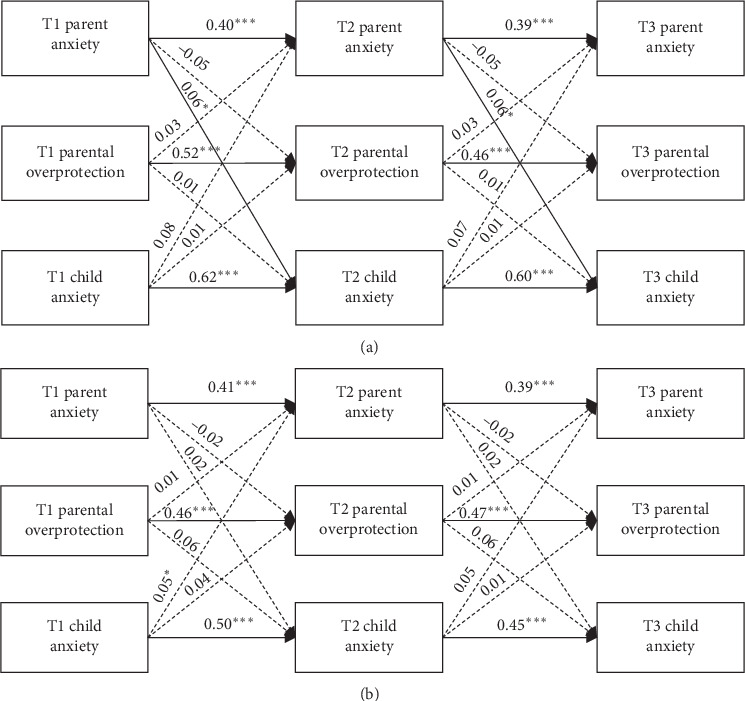
The relationship between parental overprotection and parent and child anxiety using (a) parent-reported data and (b) child-reported data. Paths represent standardized coefficients. *⁣*^*∗*^*p* < 0.034, *⁣*^*∗∗*^*p* < 0.01, and *⁣*^*∗∗∗*^*p* < 0.001. (a) includes three correlated errors (T1–T3 parent anxiety; T1–T3 parental overprotection; and T1–T3 child anxiety). (b) includes four correlated errors (T1–T3 parent anxiety; T1–T3 parental overprotection; T1–T2 child anxiety; and T1–T3 child anxiety).
